# Multicellular Co-Culture in Three-Dimensional Gelatin Methacryloyl Hydrogels for Liver Tissue Engineering

**DOI:** 10.3390/molecules24091762

**Published:** 2019-05-07

**Authors:** Juan Cui, Huaping Wang, Qing Shi, Tao Sun, Qiang Huang, Toshio Fukuda

**Affiliations:** Intelligent Robotics Institute, School of Mechatronical Engineering, Beijing Institute of Technology, 5 South Zhongguancun Street, Haidian District, Beijing 100081, China; cuijuan2016@bit.edu.cn (J.C.); shiqing8309@gmail.com (Q.S.); stnuc@sohu.com (T.S.); qhuang@bit.edu.cn (Q.H.); tofukuda@nifty.com (T.F.)

**Keywords:** GelMA hydrogel, co-culture, 3D assembly, liver tissue engineering

## Abstract

Three-dimensional (3D) tissue models replicating liver architectures and functions are increasingly being needed for regenerative medicine. However, traditional studies are focused on establishing 2D environments for hepatocytes culture since it is challenging to recreate biodegradable 3D tissue-like architecture at a micro scale by using hydrogels. In this paper, we utilized a gelatin methacryloyl (GelMA) hydrogel as a matrix to construct 3D lobule-like microtissues for co-culture of hepatocytes and fibroblasts. GelMA hydrogel with high cytocompatibility and high structural fidelity was determined to fabricate hepatocytes encapsulated micromodules with central radial-type hole by photo-crosslinking through a digital micromirror device (DMD)-based microfluidic channel. The cellular micromodules were assembled through non-contact pick-up strategy relying on local fluid-based micromanipulation. Then the assembled micromodules were coated with fibroblast-laden GelMA, subsequently irradiated by ultraviolet for integration of the 3D lobule-like microtissues encapsulating multiple cell types. With long-term co-culture, the 3D lobule-like microtissues encapsulating hepatocytes and fibroblasts maintained over 90% cell viability. The liver function of albumin secretion was enhanced for the co-cultured 3D microtissues compared to the 3D microtissues encapsulating only hepatocytes. Experimental results demonstrated that 3D lobule-like microtissues fabricated by GelMA hydrogels capable of multicellular co-culture with high cell viability and liver function, which have huge potential for liver tissue engineering and regenerative medicine applications.

## 1. Introduction

Construction of alternative liver tissues is urgently required for pharmacological and clinical research [1,2,3]. To replicate liver functions, monolayer cultures of hepatocytes are commonly used [4,5,6,7,8]. However, hepatocytes in 2D cultures lack spatial cell-cell and cell-matrix interactions, which potentially affect their phenotypic functions [9,10,11]. Since actual liver tissue is a complex three-dimensional (3D) integration of radial-structured lobules containing hepatocytes and non-parenchymal cells, engineering 3D microtissue that recapitulates lobule-like architecture and multicellular co-culture environment is essential for hepatocytes growth and functional expression [12]. In tissue engineering, hydrogels are being widely studied to construct cellular microenvironment due to their excellent similarity to the extracellular matrix (ECM) [13,14,15,16]. Many hydrogels such as alginate, gelatin, and chitosan have been exploited through various kinds of crosslinking methods for liver tissue engineering [17,18,19]. However, these studies are focused on cellular sheets constructions with simple structures, which could not mimic complex 3D tissue architectures at micro scale. In our previous study, poly(ethylene glycol) diacrylate (PEGDA) hydrogel was used to fabricate 3D constructs encapsulating hepatocytes and fibroblasts to mimic liver lobules [20]. PEGDA hydrogel has strong mechanical properties for 3D assembly, but it is non-biodegradable which limits cell organization for 3D.

Gelatin methacryloyl (GelMA) is a semi-synthetic hydrogel with biocompatibility and biodegradability. GelMA is generated by derivatization of gelatin with methacryloyl [21,22,23,24]. The gelatin component provides an aqueous environment contains many arginine-glycine-aspartic acid (RGD) sequences to support cell adhesion and proliferation [25,26]. The methacryloyl substitute groups enable photo-polymerization of GelMA in the presence of photoinitiator (PI) [27,28,29]. These features make GelMA gain increasing interest for 3D fabrication of cellular tissues [24,30,31,32]. GelMA has tunable physical properties due to the chemical modification, allowing extensive applications in various cell-laden tissues. Although many studies using GelMA have been published, they focused on fabrication of cellular constructs with simple geometry and planar assembly [24,27,33,34]. Actually, the bio-relevant geometry of the culture environment can lead to higher physiological relevance of the liver-tissue model [35]. In order to fabricate 3D tissue-mimetic microtissue with structural integrity and high cell viability, specific investigation of cross-linking condition and cell viability of GelMA is required. On the other hand, the mechanical properties are relatively weak that not suitable for contact mechanical operation. Therefore, a careful and general manipulation is needed to achieve 3D assembly and integration.

In this paper, we utilized GelMA to construct multicellular co-cultured 3D microtissues with lobule-like morphology by assembling layered cellular micromodules through local fluid-based micromanipulation. The cellular micromodules were fabricated by photo-crosslinking a mixture of GelMA and hepatocytes into hexagonal morphology with radial-type hole. Hepatocytes viability in GelMA micromodules with different degree of methacrylation were investigated to determine the optimal methacrylation. The GelMA micromodules were assembled through local fluid-based micromanipulation, which prevented GelMA from structural deformation and damage. Then assembled micromodules were coated with a mixture of GelMA and fibroblasts, subsequently irradiated by ultraviolet (UV). After that, the 3D microtissue was integrated and multiple cells encapsulation was achieved. With long-term co-culture, multiple cells encapsulated in the 3D lobule-like microtissues maintained high cell viability. Moreover, hepatocytes exhibited enhanced function of albumin secretion in co-cultured 3D microtissues than in 3D microtissues encapsulating only hepatocytes.

## 2. Results and Discussion

### 2.1. Investigation of Cross-Linking Performance and Cytocompatibility of GelMA

**Figure 1:**
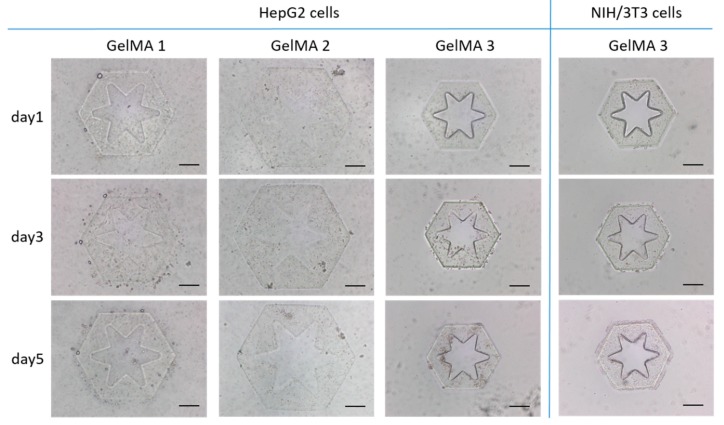
HepG2 cells and NIH/3T3 cells growth in micromodules fabricated by different kinds of GelMA. Scale bar: 200 μm.

To determine suitable GelMA for cell culture and 3D assembly, GelMA synthesized by gelatin of 10% (*w*/*v*) and methacrylic anhydride (MA) of 0.5% (*w*/*v*), 1.5% (*w*/*v*), and 20% (*w*/*v*) were prepared and named as “GelMA 1” with low degree of methacrylation, “GelMA 2” with medium degree of methacrylation, and “GelMA 3” with high degree of methacrylation. The GelMA mixed with HepG2 cells was irradiated in a microfluidic channel by UV light that came from the reflection of the digital micromirror device (DMD) illuminated by a mercury lamp. The DMD integrates micromirror array, allowing discrete reflect of UV beam to realize the shape programming of UV irradiation area. By controlling the DMD, the irradiation area of the precursor was re-shaped. So that the GelMA was cross-linked into a radial-pattern micromodule with hepatocytes encapsulation, to mimic liver lobule owing hexagonal-like geometry and radial-like pattern (Figure 1). To construct 3D lobule-like microtissue, the micromodules require to maintain their complete structures and the patterns after releasing from the microfluidic channel. For GelMA 1, one micromodule was cured by UV irradiation for about 9 s. However, the edge of the micromodule was ragged and the area of the radial pattern remained cured hydrogel. The radial-type hole is essential since it not only shortened the distance of mass transfer of encapsulated cells and culture medium, but also increased spreading area of NIH/3T3 cells after 3D assembly. For GelMA 2, the formation of one micromodule required UV irradiation for 3.5 s. In contrast, GelMA 3 was photo-crosslinked into a micromodule by only 0.5 s. Both of the GelMA 1 and GelMA 2 micromodules were obviously expanded compared to GelMA 3 micromodules after released in a dish with culture medium. The GelMA 3 kept in micro scale with more clear edge and radial-type hole to mimic liver lobule morphology.

**Figure 2:**
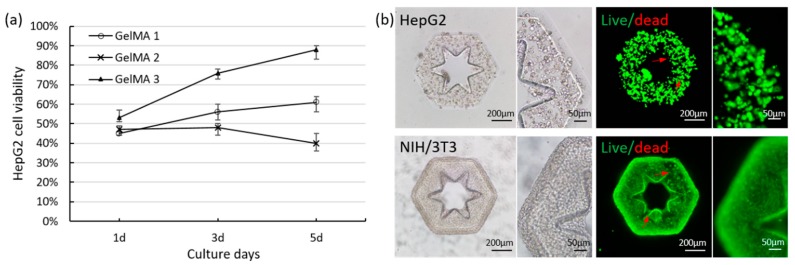
(**a**) The viability of HepG2 cells respectively encapsulated in GelMA 1, GelMA 2, and GelMA 3 during 5 days culture; (**b**) Live/dead staining of GelMA 3 micromodule encapsulating HepG2 cells and GelMA 3 micromodule encapsulating NIH/3T3 cells after culturing 10 days.

We cultured the cellular micromodules fabricated by GelMA 1, GelMA 2, and GelMA 3 for 5 days. Both HepG2 cells encapsulated in GelMA 1 and GelMA 2 have no notable proliferation in the 5 days. In GelMA 3, HepG2 cells significantly proliferated after 5 days. The live/dead assay results showed that the viability of HepG2 cells were low in the three kinds of micromodules on the first day (Figure 2a). 

The cell viability of GelMA 3 was slightly higher than that of GelMA 1 and GelMA 2 since long UV irradiation time for cross-linking GelMA 1 and GelMA 2 affected cell viabilities. With culture, cell viability of GelMA 3 increased to over 90% on the 5th day of culture, which was significantly higher than that of GelMA 1 and GelMA 2. Actually, UV irradiation not only affect cell survival during the irradiation, but also influence the ability of cell proliferation [33]. Although the viabilities of cells are comparable in the three GelMA at day 1, many cells that suffered from longer UV irradiation time no longer proliferate. Since GelMA 1 and GelMA 2 take a long UV irradiation time to cross-linking and encapsulate cells, cell viabilities of GelMA 1 and GelMA 2 exhibited lower increase rate than that of GelMA 3. Particularly, the cell viability in GelMA 1 was increased to about 60%, but in GelMA 2 was slightly decreased. The dissolved GelMA 2 solution was more viscous than GelMA 1 solution, which may affect the viability of cells mixed in GelMA 2 solution.

For multicellular co-culture, we investigated the survival of NIH/3T3 cells encapsulated in GelMA 3 micromodules. With 5 days culture, NIH/3T3 cells rapidly proliferated in GelMA 3 (Figure 1). Even culturing for 10 days, HepG2 cells and NIH/3T3 cells maintained high viability in GelMA 3 (Figure 2b). It means photo-crosslinked GelMA 3 provided a microenvironment with high cytocompatibility for cells growth. The compressive modules of micromodules by GelMA with low, medium, and high degree of methacrylation were measured (Figure S1). The GelMA with high degree of methacrylation exhibits higher compressive modulus than that of others. These results demonstrate that the GelMA 3 allowed rapid photo-crosslinking with high structural fidelity and high cell viability. Therefore, GelMA 3 fabricated cellular micromodules were selected to assemble multicellular co-cultured 3D lobule-like microtissues.

### 2.2. Long-Term Co-Culture of 3D Lobule-Like Microtissues

**Figure 3:**
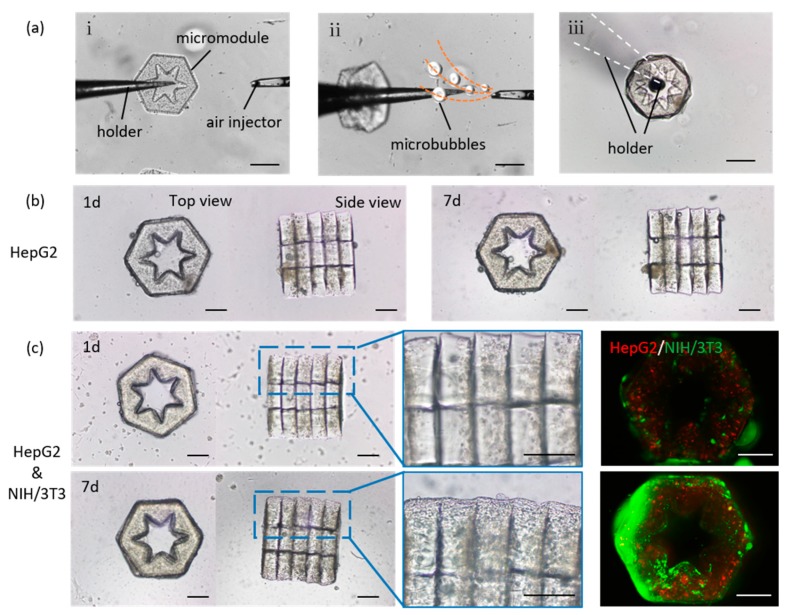
(**a**) 3D assembly process of lobule-like microtissues relying on local fluid-based micromanipulation, scale bars: 400 μm; (**b**) Long-term culture of 3D lobule-like microtissues encapsulating HepG2 cells, scale bars: 200 μm; (**c**) Long-term co-culture of 3D lobule-like microtissues encapsulating HepG2 cells and NIH/3T3 cells, scale bars: 200 μm.

The 3D construction of lobule-like microtissues was performed by assembling cellular micromodules through a local fluid-based micromanipulation strategy (Figure 3a). This strategy relying on microfluidic force, allows non-contact pick up of several GelMA micromodules (Figure S2).

For 3D construction, the picked micromodules were coated with a mixture of GelMA 3 and NIH/3T3 cells, subsequently bonded into an integration by UV irradiation. Although NIH/3T3 cells were encapsulated in the covered GelMA, it allows cell-cell interactions between HepG2 cells and NIH/3T3 cells since GelMA is a soft porous material. The HepG2 cells and NIH/3T3 cells encapsulated in the 3D lobule-like microtissues were co-cultured for 7 days. As a comparison, 3D lobule-like microtissues coating GelMA 3 without NIH/3T3 cells were constructed and cultured. With long-term culture, HepG2 cells proliferated in mono-cultured 3D microtissues (Figure 3b). For co-cultured 3D microtissues, the fluorescence results shown that both HepG2 cells and NIH/3T3 cells proliferated in the microtissues (Figure 3c). These results confirm that the 3D lobule-like microtissues fabricated by photo-crosslinked GelMA 3 provided a suitable 3D microenvironment for cell survival in vitro. Notably, in co-cultured 3D microtissue, NIH/3T3 cells proliferated and spread on the microtissue along the 3D morphology, which could improve the integrity and promote cell-cell interactions between assembled micromodules.

**Figure 4:**
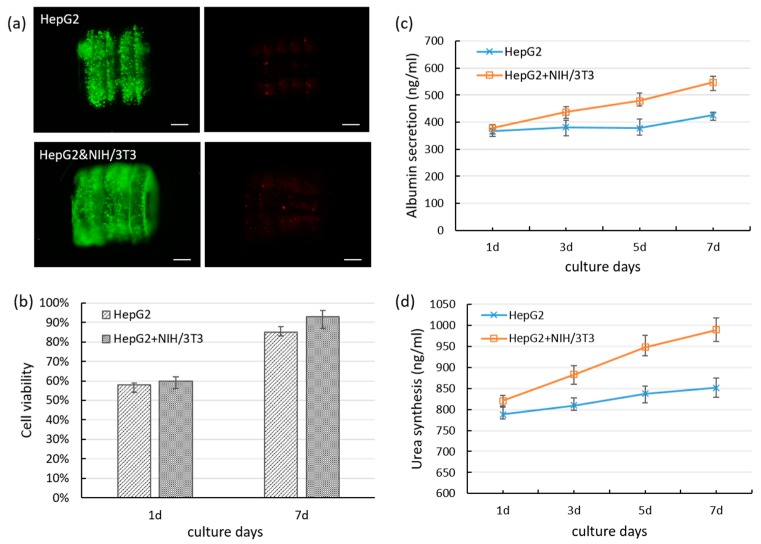
(**a**) Live/dead staining of mono-cultured 3D lobule-like microtissues and co-cultured 3D microtissues after 7 days of culture, scale bar: 200 μm; (**b**) The cell viability of mono-cultured 3D microtissues which encapsulating HepG2 cells and co-cultured 3D microtissues which encapsulating HepG2 cells and NIH/3T3 cells; (**c**) The evaluation of albumin secretion of HepG2 cells in mono-cultured 3D microtissues and co-cultured 3D microtissues during long-term culture; (**d**) The evaluation of urea synthesis of HepG2 cells in mono-cultured 3D microtissues and co-cultured 3D microtissues during culture.

After 7 days of culture, both co-cultured 3D microtissues and mono-cultured 3D microtissues were shown high cell viability (Figure 4a). It demonstrated that the cell encapsulated 3D lobule-like microtissues could maintain high cell viability in vitro. Particularly, the hole of the 3D microtissue as a vessel-like structure is essential for maintaining the viability of cells encapsulated in the hydrogel since the mass transfer effect is influenced by the distance between cells and culture medium. The radial-type hole of the 3D microtissues increased the tissue surface, which could facilitate cells spreading as well as cells contact with the oxygen and the nutrients. The quantitative results of the cell viability of the co-cultured 3D microtissues and mono-cultured 3D microtissues are shown in Figure 4b.

At the first day of culture, the cell viability of the assembled 3D microtissues were low. Cells might be damaged during the UV irradiation and assembly process, which could be optimized to minimize the influence to cells in future development of the micromanipulation system. However, the cell viability obviously increased after culturing 7 days, especially of co-cultured 3D microtissues (over 90%). The cell viability of co-cultured 3D microtissues were higher than that of mono-cultured 3D microtissues during the culture period. To investigate the function of hepatocytes in the 3D lobule-like microtissues, albumin secretions of co-cultured 3D microtissues and mono-cultured 3D microtissues were evaluated by albumin ELISA kit. As shown in Figure 4c, the albumin secretion of the co-cultured 3D microtissues were gradually increased in the long-term culture. However, the albumin secretion of the mono-cultured 3D microtissues were not significantly changed during the early culture period, and slightly increased until after 5 days. We also measured the urea synthesis of the co-cultured and mono-cultured 3D microtissues, as shown in Figure 4d. During the culture period, the co-cultured 3D microtissues maintained a higher level of urea synthesis than the mono-cultured 3D microtissues. The increase of urea synthesis in the co-culture group is approximately three times the mono-culture group. These results demonstrated that co-culture of HepG2 cells with NIH/3T3 cells in the 3D microtissues enhanced albumin secretion and urea synthesis than mono-culture of HepG2 cells in the 3D microtissues. Moreover, during the culture period, the albumin secretion and urea synthesis of the co-cultured 3D microtissues maintained higher level than that of the PEGDA-based 3D constructs in our previous work [20]. The PEGDA-based precursor solution is thicker than the GelMA one, which means cells undergo a harsh environment during the process for fabricating cellular PEGDA micromodules, thus may cause cells injury and influence cell recovery and function expression. These results indicate that GelMA-based 3D microtissues is better for maintaining cell functions than PEGDA. Actually, the micro-architecture of liver lobule is more complex. Primary hepatocytes which more relevant to liver are expected to be employed. Since the primary hepatocytes are more sensitive to damage in vitro, the fabrication process should be developed and optimized. In our future work, we expect to fabricate 3D microtissues with higher physiological relevance of liver lobule, and reconstruct heterogeneous cells to mimic radial cell-cell interactions (Figure S3).

## 3. Materials and Methods

### 3.1. Synthesis of GelMA

The synthesis of GelMA was carried by a reported method [23]. Briefly, gelatin was mixed in phosphate buffered saline (PBS, GIBCO, Gaithersburg, MD, USA) with stirring at 50 °C to form a 10% (*w*/*v*) solution. Methacrylic anhydride (MA) with the concentration of 0.5% (*w*/*v*), 1.5% (*w*/*v*), or 20% (*w*/*v*) was added to the gelatin solution at a rate of 500 μL/min, which would determine the final GelMA with low, medium and high degree of methacrylation as previously demonstrated [24]. The gelatin solution was stirred at 50 °C during the reaction and allowed to react for 1 h. After that, warm PBS was added to stop the reaction and to form a 5× dilution. The solution was dialyzed in 12–14 kDa cutoff dialysis tubing in distilled water at 40 °C for 1 week to remove extra methacrylic acid. The distilled water was changed every 12 h. The final solution was lyophilized for 1 week and then stored at −80 °C for future use.

### 3.2. Fabrication of Radial-Pattern Micromodules Encapsulating Hepatocytes

The cellular micromodules with radial pattern were fabricated relying on a DMD (ViALUX, Chemnitz, Germany)-based microfluidic channel. The device was placed on a microscope (IX81, Olympus Inc., Tokyo, Japan). The DMD served as a dynamic mask that can shape ultraviolet (UV) beams into arbitrary patterns. Before fabrication, GelMA and Irgacure 2959 photoinitiator (PI, BASF SE, Ludwigshafen, Germany) were completely dissolved in PBS at 37 °C to form a precursor solution of 10% (*w*/*v*) GelMA and 0.5% (*w*/*v*) PI. HepG2 cells (ATCC, Manassas, VA, USA) were added in the precursor and fully mixed. Then HepG2 cells or NIH/3T3 cells with concentration of 1 × 10^7^ cells·mL^−1^ were mixed with the precursor and injected into the microfluidic channel. For photo-crosslinking, UV light (25 mW/cm^2^) came from the reflection of the DMD illuminated by a mercury lamp (USH-103tems, Olympus Inc.) was focused on the precursor in the microfluidic channel through the objective lens. The irradiated area of the precursor by the re-shaped UV was cross-linked into a radial-pattern micromodule with hepatocytes encapsulation. The total dose for photo-crosslinking GelMA 1, GelMA 2, and GelMA 3 into a micromodule with complete structure are 225 mJ/cm^2^, 87.5 mJ/cm^2^, 12.5 mJ/cm^2^, respectively. By repeating UV irradiation on the uncrosslinked precursor area, numerous radial cellular micromodules were produced. After fabrication, the cellular micromodules were collected in a petri dish with culture medium. For co-culture experiments, HepG2 cells and NIH/3T3 cells were mixed at a ratio of 2:1 to form a final cell concentration of 1 × 10^7^ cells·mL^−1^, and then fully mixed with precursor for photo-crosslinking into micromodules encapsulating both HepG2 and NIH/3T3 cells.

### 3.3. 3D assembly of Lobule-Like Microtissues Encapsulating Multiple Cells

The 3D assembly was achieved by local fluid-based micromanipulation as we reported previously [36]. The system consists of a microscope and dual coordinated micromanipulators with flexible posture (Figure S4). One micromanipulator used a glass rod (G-1, Narashige International USA Inc., East Meadow, NY, USA) as the end-effector to hold the micromodules, the other micromanipulator used a glass capillary (G-1000, Narashige Inc.) as the end-effector, which was connected with a syringe pump for air injection. Both of two end-effectors were heated and pulled to get terminal diameter of 40 μm. The 3D assembly was performed in liquid. Firstly, the holder moved to the center of one micromodule. Since the center of the micromodule is hollow, the holder locked the micromodule in situ. Then air injector moved to the outside of the micromodule, and inject air to the liquid. Injected micro-bubbles generated local fluid force that lifted the micromodule up along the holder. By repeating this process, several micromodules were picked up.

After pick up, the picked micromodules need to be bonded to an integration. The holder was erected vertically to make the micromodules stacked layer by layer in the dish. The liquid was aspirated from the bottom of the dish to prevent the damage of surface tension of liquid to the GelMA micromodules. A mixture of GelMA precursor solution with NIH/3T3 cells (ATCC) was added in the dish and covered the layered micromodules. The extra precursor was removed by PBS. Then, the micromodules were wash by mineral oil. Mineral oil-GelMA interface produced hydrophilic-hydrophobic interactions which aligned the layer micromodules into a regular geometry. After that, the micromodules were irradiated by UV for 5 s to cross-link the covered cellular precursor. Finally, the HepG2 cells and NIH/3T3 cells encapsulated 3D microtissue with lobule-like geometry was performed and could be released in culture medium for long-term culture (Figure S2). 3D microtissues coating cell-free GelMA also assembled and cultured for the control treatment.

### 3.4. Evaluation of Cell Viability and Proliferation

Live/dead staining of cellular micromodules and 3D cellular microtissues were performed by using calcein AM (2 µg/mL) and propidium iodide (3 µg/mL) fluorescent stains (Molecular Probes, Eugene, OR, USA). For quantitative anaylsis of cell viability, Cell Counting Kit-8 (CCK-8, Dojindo Molecular Technologies Inc., Tokyo, Japan) was used. The absorbance of the result solution was measured using a microplate reader. To monitor cell proliferation of co-cultured 3D microtissues, HepG2 cells and NIH/3T3 cells were seperately stained before encapsulation by using PKH26 (red) and PKH67 (green) fluorescent cell linker kits (Sigma-Aldrich Chemical Co., St. Louis, MO, USA). The results were monitored with the fluorescence microscopy.

### 3.5. Evaluation of Albumin Secretion

The albumin secretion of mono-cultured 3D lobule-like microtissues which encapsulating HepG2 cells and co-cultured 3D lobule-like microtissues which encapsulating HepG2 cells and NIH/3T3 cells were respectively measured by using human albumin enzyme-linked immunosorbent assay (ELISA) kit (Sigma). The culture mediums of the mono-cultured 3D microtissues and the co-cultured 3D microtissues were respectively collected every 2 days. Then the culture mediums were centrifuged to remove the impurity, subsequently stored at −80 °C. After collection for 7 days, the mediums were gradually thawed to room temperature and added to the ELISA kit. The results were read by a microplate reader.

### 3.6. Statistical Analysis

All data were represented as means ± standard deviation (SD). Values were compared by student’s *t*-test and one-way ANOVA test followed by Bonferroni test (*p* < 0.05).

## 4. Conclusions

In summary, we have successfully demonstrated the use of GelMA hydrogels in fabricating multicellular co-cultured 3D microtissues with lobule-like structures for mimicking actual liver lobules. By photo-crosslinking GelMA through DMD-based microfluidic channel, hepatocytes encapsulated micromodules with hexagonal morphology and radial-type hole were fabricated. The micromodules with high structural fidelity allowed 3D assembly of lobule-like microtissues by local fluid-based micromanipulation through non-contact pick-up strategy. The assembled micromodules were coated with GelMA mixing with fibroblasts for integrating into 3D lobule-like microtissues through UV irradiation. The 3D microtissues encapsulating hepatocytes and fibroblasts were long-term cultured. Experimental results shown that the multicellular co-cultured 3D microtissues maintained high cell viability. Moreover, the multicellular co-cultured 3D microtissues exhibited higher level of albumin secretion than the 3D microtissues encapsulating only hepatocytes. These results indicated that the GelMA-based 3D lobule-like microtissues allow long-term co-culture of hepatocytes and fibroblasts with high cell viability and enhanced function of albumin secretion in vitro. We expect that our approach will be expanded to construct biodegradable 3D microtissues with primary hepatocytes for more physiological relevance to liver lobule, and will be potential for regenerative medicine research.

## Supplementary Materials

The following are available online at https://www.mdpi.com/1420-3049/24/9/1762/s1, Figures S1–S4.
