# Supplementary material for: Multicellular Co-Culture in Three-Dimensional Gelatin Methacryloyl Hydrogels for Liver Tissue Engineering
Source: Molecules. 2019 May 7;24(9):1762. doi: 10.3390/molecules24091762 (PMC6539120; doi:10.3390/molecules24091762)
Supplement: Supplementary file 1 [file molecules-24-01762-s001.pdf]

Supplementary Information

Communication

Multicellular co-culture in three-dimensional gelatin methacrylate hydrogels for liver tissue engineering

Juan Cui, Huaping Wang*, Qing Shi, Tao Sun, Qiang Huang and Toshio Fukuda
School of Mechatronical Engineering
Beijing Institute of Technology, Beijing 100081, P. R. China
E-mail: wanghuaping@bit.edu.cn (H. Wang)

Figure S1 The compressive modulus of GelMA-made micromodules···························2

Figure S2 3D lobule-like microtissues consist of different number of micromodules········· 3

Figure S3 Schematic demonstration of lobule-mimetic composite microstructures····· 4

Figure S4 View of local fluid-based micromanipulation system·································

5

Supplementary Figures

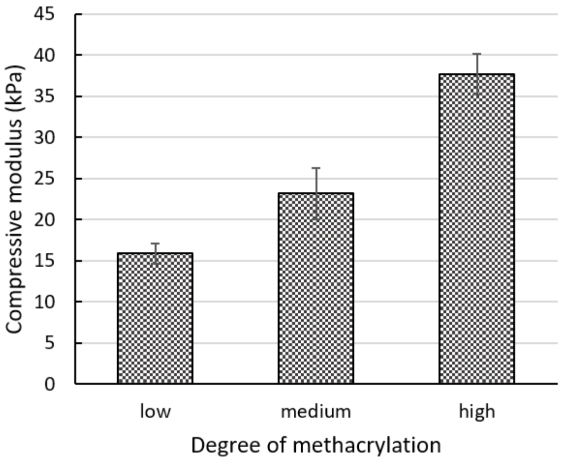

Figure S1. The compressive modulus of micromodules fabricated by GelMA with low, medium, and high degree of methacrylation.

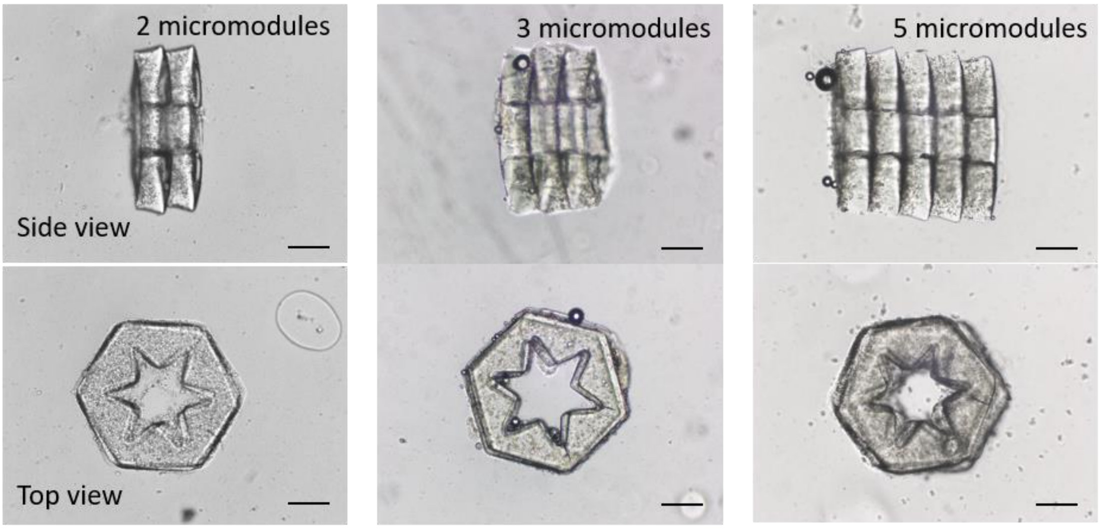

Figure S2. 3D lobule-like microtissues consist of different numbers of micromodules, scale bar: 200 μm.

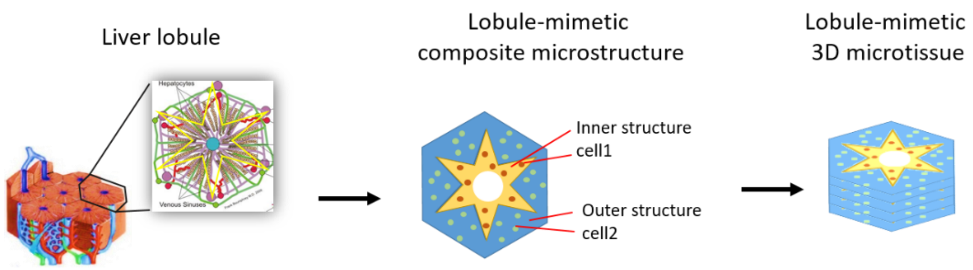

Figure S3. Schematic demonstration of lobule-mimetic composite microstructures.

As shown in Figure S3, the 3D lobule-mimetic microtissue which consistes of composite microstructures contain two parts: inner structure encapsulating one kinds of cells, and outer structure encapsulating another kinds of cells. The outer structure can mimic the hexagonal morphology of liver lobule, and the inner radial structure can mimic the inner radiating distribution of liver lobule. We hope the inner radiating morphology can promote cell-cell interactions and ensure liver functions.

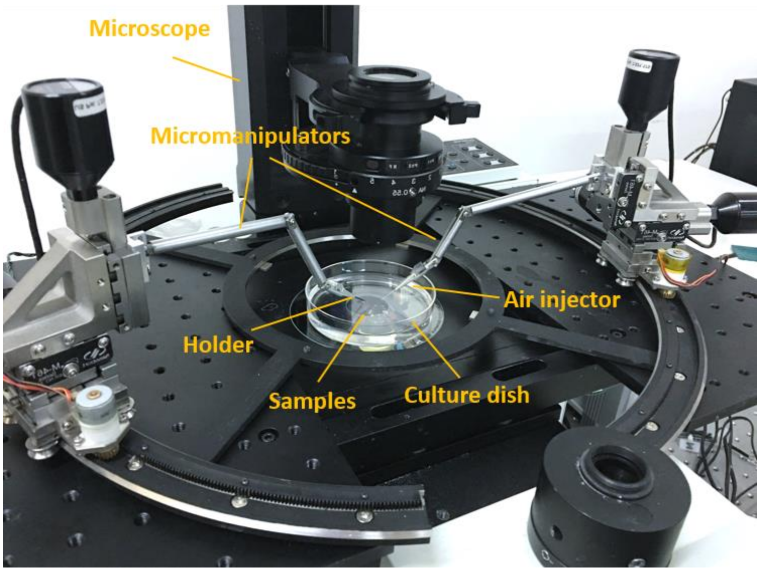

Figure S4. Local fluid-based manipulation system for 3D assembly of lobule-like microtissues. The system consists of dual micromanipulators and a rail. The micromanipulators carry on three piezo motors for micro-scale translation with three degrees of freedoms, and are driven by stepping motor for circular motion on rail. The pulled glass rod and glass capillary as end-effectors are separately fixed on the micromanipulators for operation of micro-scale modules. The rail supports circular motion trajectory of dual micromanipulators to restrict the end-effectors moving in the microscope vision. The manipulation system employs the vision feedback system to realize automatic recognition and location of objects and end-effectors.
